# Complete Genome Sequence of Salmonella enterica Serovar Heidelberg Siphophage Sepoy

**DOI:** 10.1128/MRA.00680-19

**Published:** 2019-07-25

**Authors:** Armando M. Marrufo, Chi Zeng, Chandler O’Leary, Lauren Lessor, Rohit Kongari, Jason Gill, Mei Liu

**Affiliations:** a Center for Phage Technology, Texas A&M University, College Station, Texas, USA; b School of Biology and Pharmaceutical Engineering, Wuhan Polytechnic University, Wuhan, China; Loyola University Chicago

## Abstract

Phage Sepoy infects Salmonella enterica serovar Heidelberg, a Gram-negative bacterium that causes severe foodborne illnesses. Bacteriophages infecting this pathogen may be used as biocontrol agents for preventing Salmonella foodborne diseases. Here, we present the complete genome sequence of Sepoy, a T5-like siphophage.

## ANNOUNCEMENT

Salmonellosis is a common foodborne illness caused by the consumption of food products contaminated with Salmonella (1). With the rise of new serotypes and antimicrobial resistance, there is a need to study alternative biocontrol approaches to prevent *Salmonella* foodborne diseases. The potential of phage therapy to limit food contamination is being explored in the poultry industry (2, 3). Here, we present and describe the features of the complete genome of Sepoy, a T5-like siphophage that infects Salmonella enterica serovar Heidelberg.

Siphophage Sepoy was isolated from a wastewater treatment plant in College Station, TX, in 2015 using *Salmonella* Heidelberg as a host. Host bacteria were cultured on tryptic soy broth or agar (Difco) at 37°C with aeration. Phage was cultured and propagated by the soft-agar overlay method (4) and was identified as a siphophage using negative-stain transmission electron microscopy performed at the Texas A&M University Microscopy and Imaging Center, as described previously (5). Phage genomic DNA was prepared using a modified Promega Wizard DNA clean-up kit protocol, as described previously (5). Pooled indexed DNA libraries were prepared using the Illumina TruSeq Nano low-throughput (LT) kit, and the sequence was obtained from the Illumina MiSeq platform using the MiSeq V2 500-cycle reagent kit, following the manufacturer’s instructions, producing 597,167 paired-end reads for the index containing the phage Sepoy genome. FastQC 0.11.5 (https://www.bioinformatics.babraham.ac.uk/projects/fastqc/) was used to quality control the reads. The reads were trimmed with FASTX-Toolkit 0.0.14 (http://hannonlab.cshl.edu/fastx_toolkit/download.html) before being assembled using SPAdes 3.5.0 (6). Contig completion was confirmed by PCR using primers (5′-TACGCAAGGTAGTCGAGCAA-3′, 5′-GCAGTTTCCGCAACAACAAT-3′) facing off the ends of the assembled contig and Sanger sequencing of the resulting product, with the contig sequence manually corrected to match the resulting Sanger sequencing read. GLIMMER 3.0 (7) and MetaGeneAnnotator 1.0 (8) were used to predict protein-coding genes with manual verification, and tRNA genes were predicted with ARAGORN 2.36 (9). Rho-independent termination sites were identified via TransTerm (http://transterm.cbcb.umd.edu/). Sequence similarity searches were done by BLASTp 2.2.28 (10) against the NCBI nr, UniProt, Swiss-Prot (11), and TrEMBL databases. InterProScan 5.15-54.0 (12), LipoP (13), and TMHMM v2.0 (14) were used to predict protein function. All analyses were conducted at default settings via the CPT Galaxy (15) and Web Apollo (16) interfaces (https://cpt.tamu.edu/galaxy-pub).

Sepoy was assembled at 22.5-fold coverage into a 114,080-bp genome with a 10,295-bp direct terminal repeat as determined by PhageTerm (17). The GC content of Sepoy is 40.6%. Sepoy has 25 tRNAs and 185 predicted protein-coding genes, among which 62 code for proteins with a putative function. Like phage T5 (18), the Sepoy genome can be broadly divided into the following three parts: the preearly region, which is involved in early transfer of phage DNA into the host; the early region, which is responsible for DNA metabolism; and the late region, which consists of morphogenesis and lysis genes. Sepoy encodes a class III holin, an l-alanoyl-d-glutamate peptidase, and an overlapping spanin pair. Sepoy shares 89% and 67% overall DNA sequence identity with *Salmonella* phage Stitch (GenBank accession no. KM236244) (19) and phage T5 (GenBank accession no. AY543070), respectively, as determined by BLASTn.

### Data availability.

The genome sequence of phage Sepoy was submitted to GenBank as accession no. MK728825. The associated BioProject, SRA, and BioSample accession numbers are PRJNA222858, SRR8761742, and SAMN11191518, respectively.
